# Knockdown of PKM2 and GLS1 expression can significantly reverse oxaliplatin-resistance in colorectal cancer cells

**DOI:** 10.18632/oncotarget.17396

**Published:** 2017-04-24

**Authors:** Wei-Qun Lu, Ying-Ying Hu, Xiao-Ping Lin, Wei Fan

**Affiliations:** ^1^ Department of Nuclear Medicine, Sun Yat-Sen University Cancer Center, State Key Laboratory of Oncology in South China, Collaborative Innovation Center for Cancer Medicine, Guangzhou, P.R. China

**Keywords:** colorectal cancer, pyruvate kinase M2 type (PKM2), kidney-type glutaminase (GLS1), knockdown, expression

## Abstract

Clinical treatment for colorectal cancer (CRC) thus far encounters a huge challenge due to oxaliplatin-resistance. As crucial rate-limiting enzymes in aerobic glycolysis and glutaminolysis, pyruvate kinase M2 type (PKM2) and kidney-type glutaminase (GLS1) are proposed to carry important implications in colorectal carcinogenesis and drug-resistance. This study aimed to explore the possible association of oxaliplatin-resistance with aerobic glycolysis/glutaminolysis indexed by PKM2/GLS1 expression. PKM2 and GLS1 expression was quantified by polymerase chain reaction (PCR) and Western blot techniques in CRC cell lines. The abilities of cell formation, kinetics, migration, invasion, survival and apoptosis, as well as permeability glycoprotein (Pgp) expression were inspected before and after knocking-down PKM2/GLS1 expression. In addition, the influence of knocking-down PKM2/GLS1 expression was evaluated *in vivo*. Differentiated PKM2 and GLS1 expression in both THC8307 and THC8307/Oxa cell lines was identified. In the THC8307 cell line, PKM2 and GLS1 can accelerate malignant behaviors, increase oxaliplatin-resistance, upregulate Pgp expression, and inhibit cell apoptosis. Contrastingly in the THC8307/Oxa cell line, knockdown of PKM2/GLS1 expression can restrain malignant behaviors, reestablish oxaliplatin-sensitivity, downregulate Pgp expression, and induce cell apoptosis. In xenograft, knockdown of PKM2/GLS1 expression can significantly inhibit tumor growth, reduce Pgp expression, and increase tumor apoptosis. Taken together, the present findings enriched our knowledge by demonstrating a significant association of PKM2 and GLS1 with oxaliplatin-resistance in CRC. We further propose that knockdown of PKM2/GLS1 expression may constitute a novel therapeutic strategy toward effective treatment for CRC.

## INTRODUCTION

Colorectal cancer (CRC) ranks as the third most common cancer around the world. Globally, over 1 million people are newly diagnosed to develop CRC per annum [1]. In China, the incidence of CRC is rapidly escalating, especially in underdeveloped areas [2]. Currently, oxaliplatin-based chemotherapy is a chief therapeutic strategy for CRC, and it receives an intense attention in the medical literature [3]. However, oxaliplatin-resistance thus far poses a huge challenge for CRC treatment in routine clinical practice, and the understanding of molecular mechanisms underlying chemotherapy resistance of oxaliplatin is still poor [4–7].

It is widely recognized that some alterations are necessitated for cancer cells to adapt in response to chemotherapeutic stress for survival [8]. Also, there are several existing contributors to oxaliplatin-resistance, and ATP is persistently involved in almost all processes [9]. For instance, Zhou et al. reported that intracellular ATP concentration was a critical determinant of chemoresistance of oxaliplatin in colon cancer cells [10]. Chemotherapy sensitivity of drug-resistant cells could be elevated after consuming ATP. In contrast, chemotherapy resistance of drug-sensitive cells might be augmented after supplying exogenetic ATP [10]. Generally, there are two major sources of ATP in tumor cells: aerobic glycolysis and glutamine metabolism [11]. For aerobic glycolysis, pyruvate kinase (PK) is a key kinase, and PKM2 is one of PK's four isoforms. PKM2 is proposed to play a key role in cancer metabolism and account for the “Warburg” effect [12]. Experimental data are accumulating suggesting that PKM2 possesses multiple non-metabolic functions during carcinogenesis and chemoresistance [13]. For glutamine metabolism, a growing number of studies have underscored the close relationship between kidney-type glutaminase (GLS1) expression and human cancer [14–16]. Moreover, GLS1 was also found to be highly associated with drug-resistance. Fu et al. found that downregulating GLS1 could re-sensitize the Taxol-resistant breast cancer cells to Taxol [17]. Moreover, Guo et al. observed that inhibiting GLS1 could dramatically sensitize the PP242-induced cell death in ovarian cancer, and substantially reduce the phosphorylated STAT3 expression [18].

To explore the possible association of oxaliplatin-resistance with aerobic glycolysis and glutamine metabolism, we sought to employ small interfering RNA (siRNA) to knockdown the expression of PKM and GLS1. Then, malignant behaviors, 50% inhibiting concentration (IC50), permeability glycoprotein (Pgp) expression, and cell apoptosis *in vitro* and *in vivo* were observed and tested, striving to elucidate the potential molecular mechanisms of oxaliplatin-resistance in CRC cells.

## RESULTS

### Differentiated PKM2/GLS1 expression in CRC cell lines

**Figure 1:**
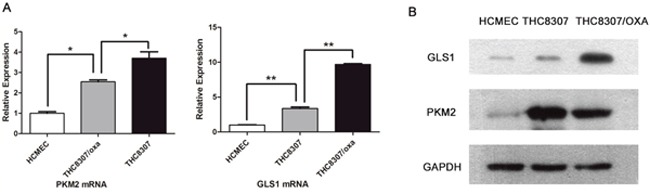
**(A)** The qRT-PCR technique showed differentiated expression of PKM2 and GLS1 in CRC cell lines. PKM2 mRNA expression in the THC8307 and THC8307/Oxa cell lines were 3.7 ± 0.53 (mean ± standard deviation) and 2.56 ± 0.11 folded higher than that in the HCMEC cell line (*p < 0.05). PKM2 mRNA expression in the THC8307 cell line was also higher than that in the THC8307/Oxa cell line (*p < 0.05). GLS1 mRNA expression in the THC8307 and THC8307/Oxa cell lines were 3.34 ± 0.38 and 9.69 ± 0.17 folded higher than that in the HCMEC cell line (**p < 0.01). By contrary with PKM2, GLS1 mRNA expression in the THC8307 cell line was lower than that in the THC8307/Oxa cell (**p < 0.01). **(B)** Compared with the HCMEC, PKM2 and GLS1 expression was elevated in two CRC cell lines - THC8307 and THC8307/Oxa). PKM2 expression was higher in the THC8307 cell line than that in the THC8307/Oxa cell line, while GLS1 expression was higher in the THC8307/Oxa cell line than that in the THC8307 cell line.

As indicated by qRT-PCR (quantitative reverse transcription - polymerase chain reaction) technique, PKM2 mRNA expression was 3.7 ± 0.53 (mean ± standard deviation, similarly hereinafter) and 2.56 ± 0.11 in CRC cell line (THC8307) and in oxaliplatin-resistant CRC cell line (THC8307/Oxa), respectively, as compared with that in the HCMEC cell line. PKM2 expression was significantly higher in the THC8307 cell line than in the THC8307/Oxa cell line, and it was also significantly higher in the THC8307/Oxa cell line than in the HCMEC cell line (both p < 0.05). Similarly, GLS1 mRNA expression was 3.34 ± 0.38 and 9.69 ± 0.17 in the THC8307 and THC8307/Oxa cell lines respectively, as compared with that in the HCMEC cell line, the differences being statistically significant (both p < 0.05) (Figure 1A). Subsequently, protein expression quantified by Western blot (WB) technique further confirmed the differentiated expression of GLS1 and PKM2 in CRC cell lines (Figure 1B).

### Knockdown of PKM2/GLS1 expression

**Figure 2:**
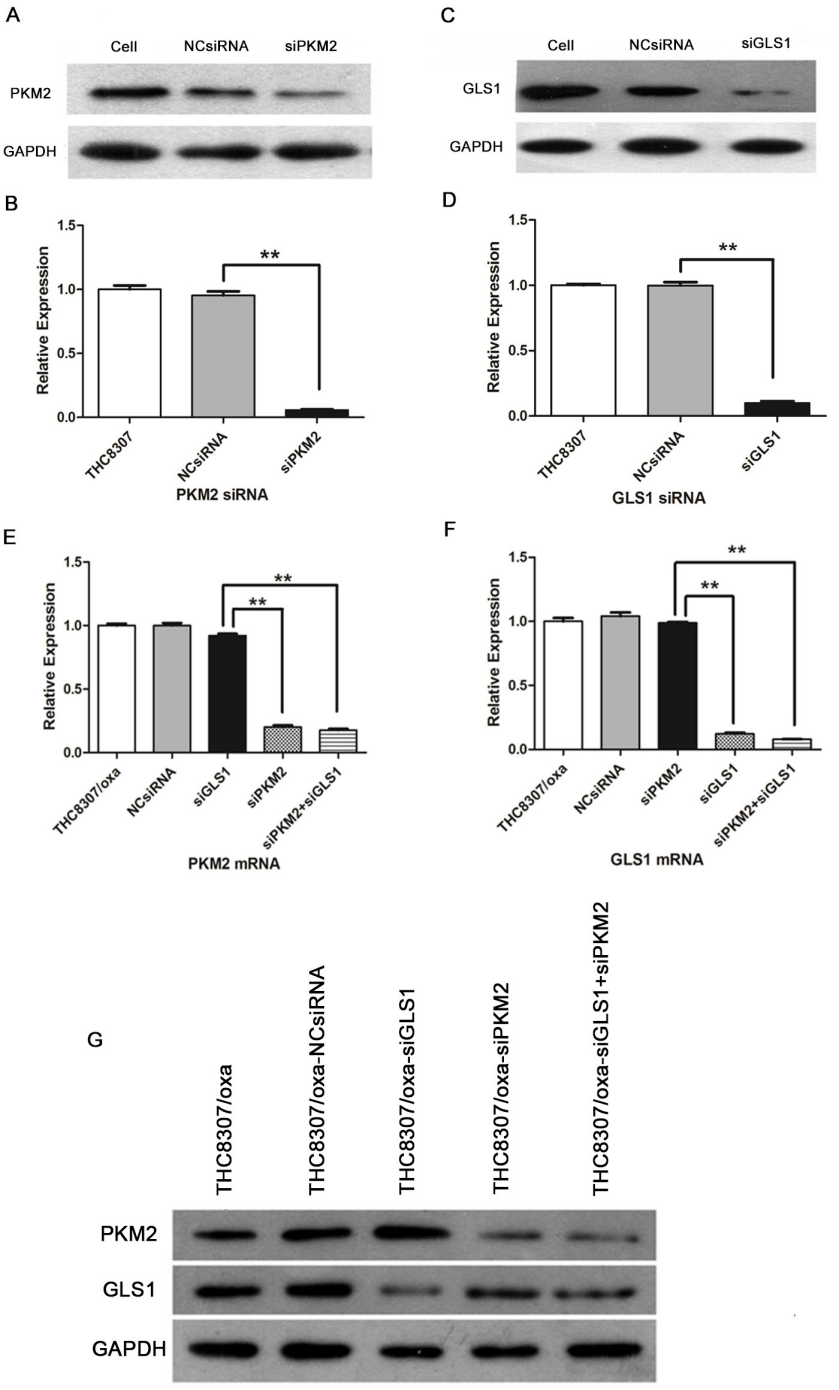
Evaluation of siRNA effectiveness in CRC cell lines (THC8307 and THC8307/Oxa) **(A-B)** WB technique showed that PKM2 expression was significantly inhibited after PKM2-siRNA (siPKM2) in the THC8307 cell line. **(C-D)** WB technique showed that GLS1 expression was significantly inhibited after GLS1-siRNA (siGLS1) in the THC8307 cell line. The expression of PKM2/GLS1 was remarkably decreased in the siRNA group, compared with that in the NCsiRNA group (**p < 0.01). **(E-F)** qRT-PCR technique confirmed PKM2/GLS1 mRNA expression was significantly inhibited after PKM2-siRNA (siPKM2)/GLS1-siRNA (siGLS1) in the THC8307/Oxa cell line, which did not affect GLS1/PKM2 mRNA expression (**p < 0.01). The combination of PKM2-siRNA with GLS1-siRNA achieved the optimal siRNA efficiency in the downregulation of PKM2/GLS1 (**p < 0.01). **(G)** WB technique confirmed the siRNA efficiency in PKM2/GLS1 downregulation in the THC8307/Oxa cell line.

After siRNA transfection, PKM2/GLS1 expression was further confirmed in the THC8307 and THC8307/Oxa cell lines. As shown in Figure 2A, 2B, 2C and 2D, PKM2/GLS1 expression was successfully inhibited in the THC8307 cell line, as shown by WB technique. Simultaneously, in the THC8307/Oxa cell line, PKM2/GLS1 expression was decreased based on qRT-PCR and WB techniques, especially in the siPKM2+siGLS1 group. Moreover, no interference phenomena appeared in two siRNAs, and the THC8307/Oxa cells were qualified to carry out subsequent investigations after knocking-down PKM2/GLS1 expression (Figure 2E, 2F and 2G).

### Malignant behaviors of CRC cell lines

**Figure 3:**
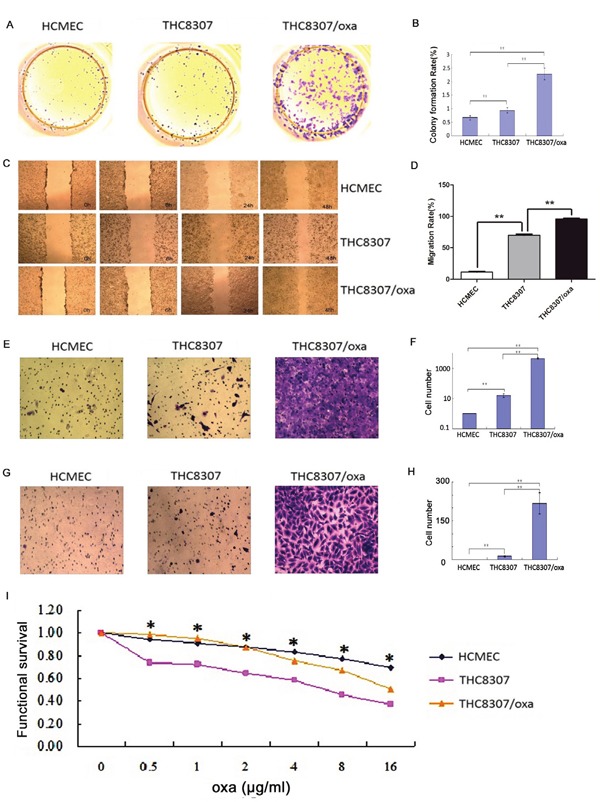
**(A-B)** Cell formation values in the HCMEC, THC8307 and THC8307/Oxa cells were 0.66 ± 0.07, 0.94 ± 0.09 and 2.28 ± 0.21, respectively (**p < 0.01). **(C-D)** The THC8307/Oxa cell line exhibited the most significant wound healing ability, as compared with that in the THC8307 and HCMEC cell lines (**p < 0.01). **(E-F)** Cell numbers in the HCMEC, THC8307 and THC8307/Oxa cells were 0 ± 0, 16 ± 4.29 and 4445.17 ± 517.45, respectively (**p < 0.01). **(G-H)** Cell numbers in the HCMEC, THC8307 and THC8307/Oxa cell lines were 0 ± 0, 12.8 ± 3.03 and 218 ± 41.31, respectively (**p < 0.01). **(I)** Cell survival rate in the THC8307 cell line was critically inhibited with the increase in oxaliplatin concentration. In comparison, the THC8307/Oxa cell line displayed a higher survival rate than that in the THC8307 cell line (*p < 0.05).

Colony formation, wound healing, Transwell test, MTS test and IC50 calculation were performed in both THC8307 and THC8307/Oxa cell lines. Before knocking-down PKM2/GLS1 expression, the THC8307/Oxa cell line exhibited the most significant cell formation ability (Figure 3A, 3B), wound healing ability (Figure 3C, 3D), cell migration ability (Figure 3E, 3F) and cell invasion ability (Figure 3G, 3H), as compared with the THC8307 and HCMEC cell lines (p < 0.05). Drug resistance examined by MTS showed that with the increase of oxaliplatin concentration, cell survival rate in the THC8307 cell line was critically inhibited. In contrast, the THC8307/Oxa cell line had a higher survival rate than the THC8307 cell line (Figure 3I). The following results on IC50 illustrated that the THC8307/Oxa cell line had significant higher IC50 than the THC8307 cell line, as presented in Table 1.

**Table 1:** IC50 value to oxaliplatin in three cell lines

Cell lines	IC50 (μg/mL)
HCMEC	324.86
THC8307	5.97
THC8307/Oxa	22.63

**Figure 4:**
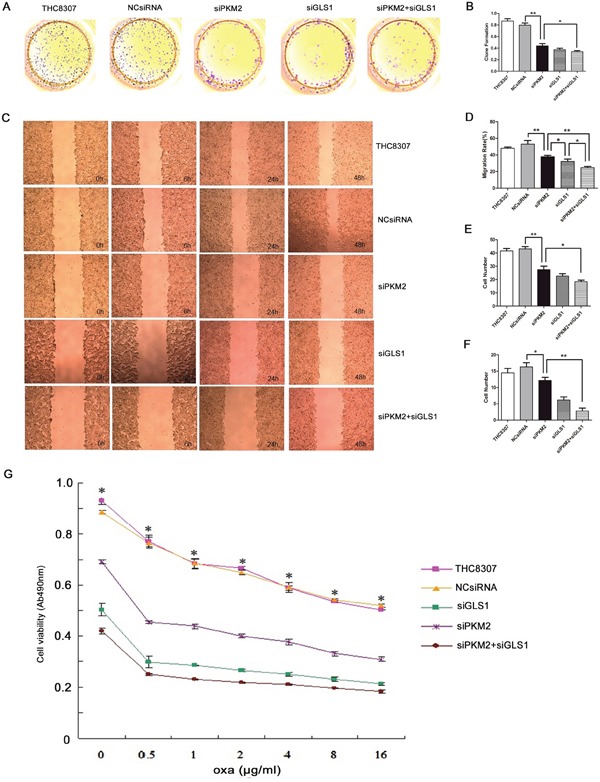
Evaluation of malignant behaviors after knocking-down PKM2/GLS1 expression in the THC8307 cell line **(A-B)** Cell formation values in the THC8307, THC8307/NCsiRNA, THC8307/siPKM2, THC8307/siGLS1 and THC8307/siPKM2 plus siGLS1 were 0.87 ± 0.12, 0.8 ± 0.11, 0.44 ± 0.11, 0.38 ± 0.07 and 0.34 ± 0.05, respectively (*p < 0.05, **p < 0.01). **(C-D)** The siPKM2 + siGLS1 group showed a substantial inhibitory efficiency of wound healing ability (*p < 0.05, **p < 0.01). **(E)** Cell numbers in the THC8307, THC8307/NCsiRNA, THC8307/siPKM2, THC8307/siGLS1 and THC8307/siPKM2 plus siGLS1 were 41.32 ± 3.9, 43.13 ± 3.75, 27.60 ± 5.99, 22.85 ± 3.88 and 18.46 ± 2.87, respectively (*p < 0.05, **p < 0.01). **(F)** Cell numbers in the THC8307, THC8307/NCsiRNA, THC8307/siPKM2, THC8307/siGLS1 and THC8307/siPKM2 plus siGLS1 were 14.51 ± 2.94, 16.47 ± 2.89, 10.38 ± 2.4, 5.88 ± 2.09 and 2.97 ± 1.89, respectively (*p < 0.05, **p < 0.01). **(G)** MTS test showed that cell survival rate in the siPKM2 plus siGLS1 group of THC8307 was dramatically inhibited with the increase in oxaliplatin concentration, as compared with the other treatment groups (*p < 0.05).

**Figure 5:**
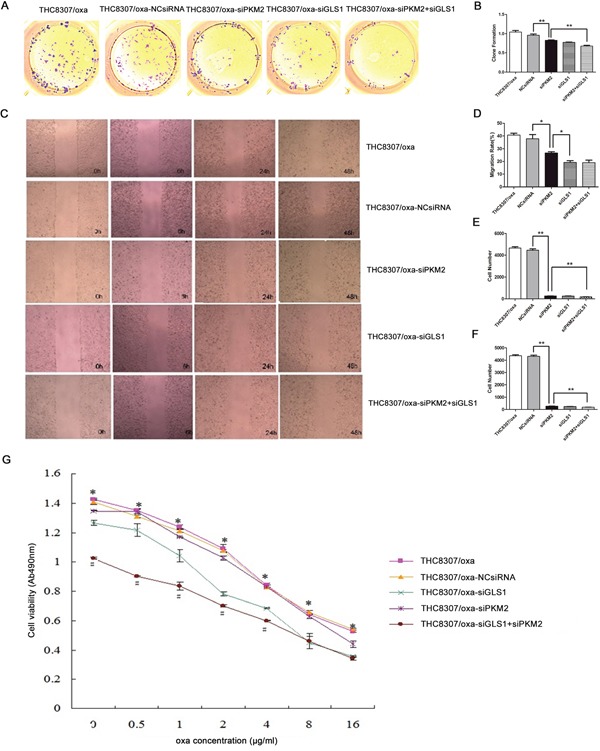
Evaluation of malignant behaviors after knocking-down PKM2/GLS1 expression in the THC8307/Oxa cell line **(A-B)** Cell formation values in the THC8307/Oxa, THC8307/Oxa-NCsiRNA, THC8307/Oxa-siPKM2, THC8307/Oxa-siGLS1 and THC8307/Oxa-siPKM2 plus siGLS1 were 1.04 ± 0.12, 0.96 ± 0.11, 0.83 ± 0.03, 0.78 ± 0.02 and 0.68 ± 0.06, respectively (**p < 0.01). **(C-D)** The siPKM2 plus siGLS1 group showed a substantial inhibitory efficiency of wound healing ability (*p < 0.05). **(E)** Cell numbers in the THC8307/Oxa, THC8307/Oxa-NCsiRNA, THC8307/Oxa-siPKM2, THC8307/Oxa-siGLS1 and THC8307/Oxa-siPKM2 plus siGLS1 were 4163.35 ± 189.89, 4316.85 ± 208.39, 268.43 ± 12.13, 252.02 ± 29.77 and 175.47±19.35, respectively (**p < 0.01). **(F)** Cell numbers in the THC8307/Oxa, THC8307/Oxa-NCsiRNA, THC8307/Oxa-siPKM2, THC8307/Oxa-siGLS1 and THC8307/Oxa-siPKM2 plus siGLS1 were 465.33 ± 263.37, 445.85 ± 287.37, 266.45 ± 21.18, 238.80 ± 23.25 and 181.60 ± 17.35, respectively (**p < 0.01). **(G)** MTS test showed that cell survival rate in the THC8307/Oxa-siPKM2 plus siGLS1 group was dramatically inhibited with the increase in oxaliplatin concentration, as compared with the other treatment groups (*p < 0.05).

After knocking-down PKM2/GLS1 expression in the THC8307 and THC8307/Oxa cell lines, the substantial inhibitory efficiencies of cell formation ability (Figure 4A, 4B, 5A and 5B), wound healing ability (Figure 4C, 4D, 5C and 5D), cell migration ability (Figure 4E and 5E) and cell invasion ability (Figure 4F and 5F) were identified in the siPKM2+siGLS1 group, as compared with the other treatment groups. Similarly, MTS test demonstrated that cell survival rate in the siPKM2+siGLS1 group was dramatically inhibited in the THC8307 and THC8307/Oxa cell lines (Figure 4G and 5G). Correspondingly, the siPKM2+siGLS1 group in the THC8307 and THC8307/Oxa cell lines exhibited the lowest IC50 (Table 2 and Table 3).

**Table 2:** IC50 value to oxaliplatin in different siRNA treatment groups after knocking-down PKM2/GLS1 expression in the THC8307 cell line

Treatment groups	IC50 (μg/mL)
THC8307	7.37
THC8307/NCsiRNA	7.09
THC8307/siPKM2	5.55
THC8307/siGLS1	4.60
THC8307/siGLS1 plus siPKM2	4.05

**Table 3:** IC50 value to oxaliplatin in different siRNA treatment groups after knocking-down PKM2/GLS1 expression in the THC8307/Oxa cell line

Treatment groups	IC50 (μg/mL)
THC8307/Oxa	25.37
THC8307/Oxa-NCsiRNA	27.09
THC8307/Oxa-siPKM2	24.95
THC8307/Oxa-siGLS1	3.69
THC8307/Oxa-siPKM2 plus siGLS1	2.52

### Pgp expression by immunofluorescence assay

**Figure 6:**
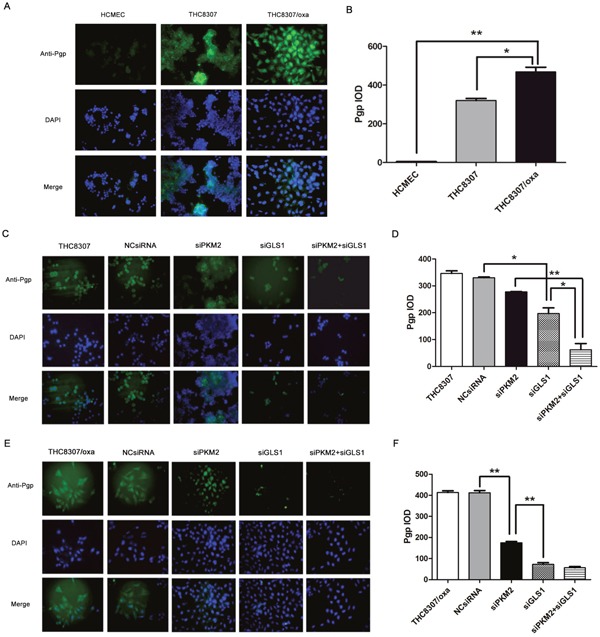
Pgp expression by immunofluorescence assay **(A-B)** The THC8307/Oxa cell line showed more Pgp expression (468.13 ± 42.13), as compared with that of the HCMEC (4.80 ± 0.88) and THC8307 (320.18 ± 18.15) cell lines (*p < 0.05, **p < 0.01). **(C-D)** Pgp expression in the THC8307, NCsiRNA, siPKM2, siGLS1 and siPKM2 plus siGLS1 were 346.29 ± 16.29, 330.32 ± 4.13, 277.42 ± 2.43, 196.83 ± 36.85 and 62.32 ± 39.62, respectively (*p < 0.05, **p < 0.01). **(E-F)** Pgp expression in the THC8307/Oxa, NCsiRNA, siPKM2, siGLS1 and siPKM2 plus siGLS1 were 406.81 ± 18.36, 413.69 ± 14.37, 174.48 ± 12.37, 71.5 ± 13.5 and 57.15 ± 11.32, respectively (**p < 0.01).

Immunofluorescence assay confirmed that the THC8307/Oxa cell line exhibited more Pgp expression (Figure 6A, 6B). After knocking-down PKM2/GLS1 expression in the THC8307 and THC8307/Oxa cell lines, Pgp expression was significantly decreased in the siPKM2+siGLS1 group (Figure 6C, 6D, 6E and 6F).

### Annexin V/PI assay

**Figure 7:**
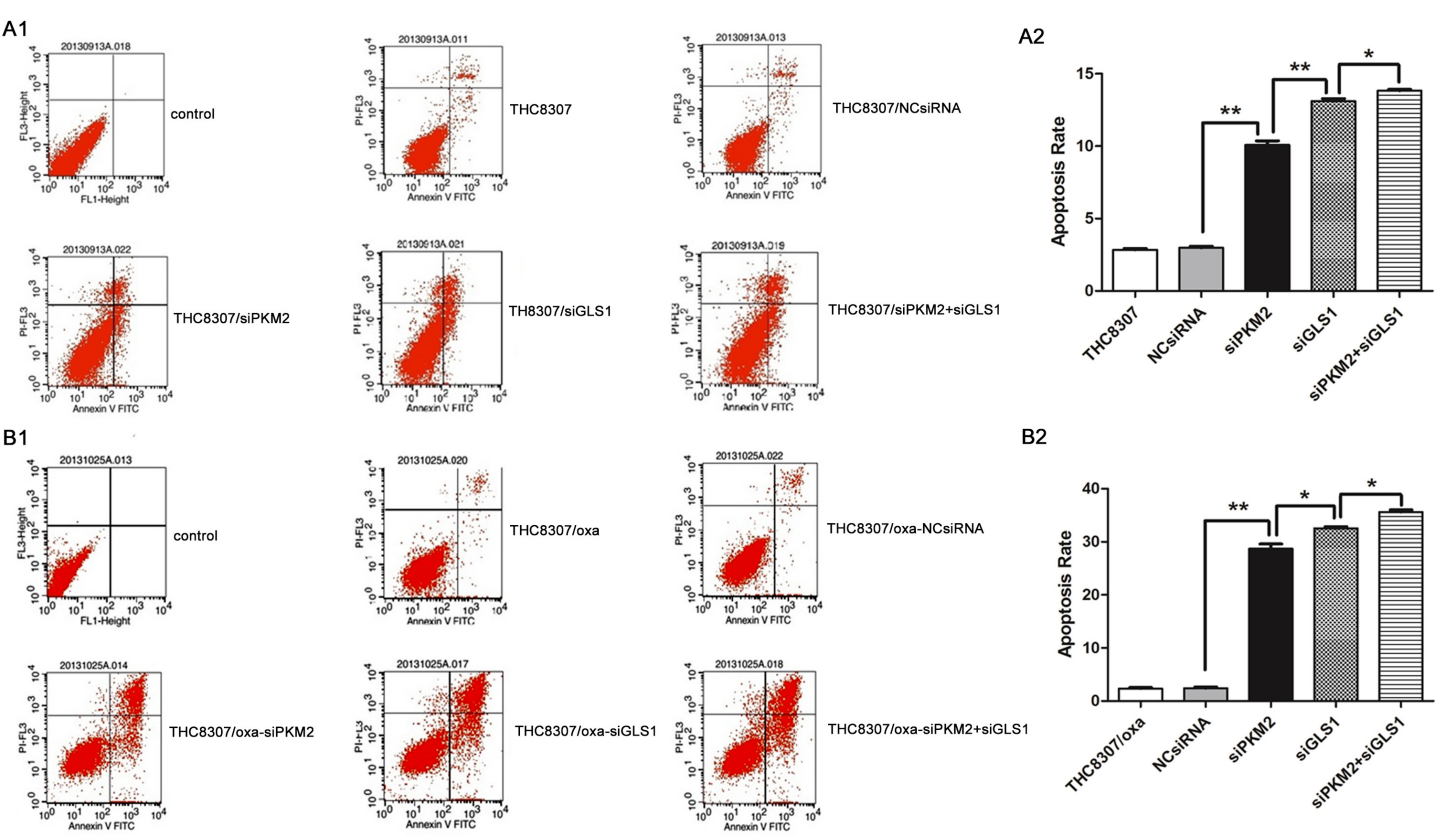
Apoptosis rate by annexin V/PI assay **(A1, A2, B1, B2)** The THC8307/Oxa cell line (apoptosis rate: 1.98 ± 0.28) possessed more anti-apoptosis ability, as compared with that in the THC8307 cell line (apoptosis rate: 3.75 ± 0.12). **(A2)** The apoptosis rate in the THC8307, NCsiRNA, siPKM2, siGLS1 and siPKM2 plus siGLS1 were 2.85 ± 0.12, 2.98 ± 0.15, 10.08 ± 0.4, 13.11 ± 0.25 and 13.83 ± 0.13, respectively (*p < 0.05, **p < 0.01). **(B2)** Apoptosis rate in the THC8307/Oxa, NCsiRNA, siPKM2, siGLS1 and siPKM2 plus siGLS1 were 2.36 ± 0.28, 2.43 ± 0.33, 29.01 ± 3.24, 34.56 ± 2.48 and 35.61 ± 0.56, respectively (*p < 0.05, **p < 0.01).

Annexin V/PI assay revealed that the THC8307/Oxa cell line (apoptosis rate: 1.98 ± 0.28) had a more anti-apoptosis ability, as compared with the THC8307 cell line (apoptosis rate: 3.75 ± 0.12). After knocking-down PKM2/GLS1 expression in the THC8307 and THC8307/Oxa cell lines, apoptosis rate was significantly boosted in the esiPKM2+siGLS1 group (Figure 7A1, A2, B1 and B2).

### Knockdown of PKM2/GLS1 expression inhibits xenograft growth

**Figure 8:**
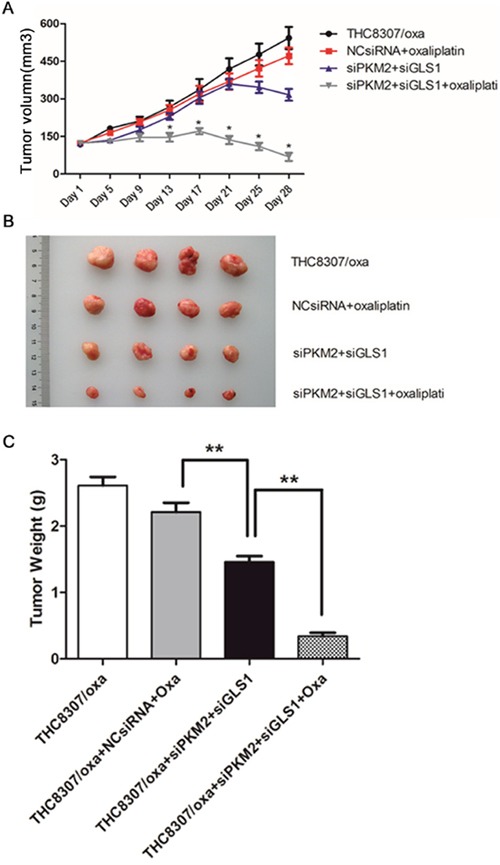
**(A-B)** Measurement of tumor volume of xenograft tumors. At the beginning, the tumors of 4 groups grew in a similar tendency, while tumor growth was significantly retarded in the siPKM2 plus siGLS1 plus oxaliplatin group at the 17^th^ day. At the 28^th^ day, the mean volume in the siPKM2 plus siGLS1 plus oxaliplatin group was intensely smaller than that in the other three xenograft groups (*p < 0.05). **(C)** Tumor weight in the siPKM2 plus siGLS1 plus oxaliplatin group was significantly reduced than that in the other three xenograft groups (**p < 0.01).

Four different groups of CRC xenograft were successfully established using the THC8307/Oxa cells, that is, THC8307/Oxa negative control (group I); NcsiRNA (100 μg/0.02 mL) plus oxaliplatin (5 mg/kg) (group II); siPKM2 (100 μg/0.02 mL) plus siGLS1 (100 μg/0.02 mL) (group III); siPKM2 (100 μg/0.02 mL) plus siGLS1 (100 μg/0.02 mL) plus oxaliplatin (5 mg/kg) (group IV). At the beginning, the tumors of 4 groups grew in a similar tendency, while tumor growth was retarded significantly in group IV at the 17^th^ day (Figure 8A). Consistently, at the 28^th^ day, the mean volume and weight of tumors in group IV were intensely smaller than that in the other three xenograft groups (Figure 8A, 8B and 8C).

### Detection of PKM2, GLS1 and Pgp in xenograft by IHC analyses

**Figure 9:**
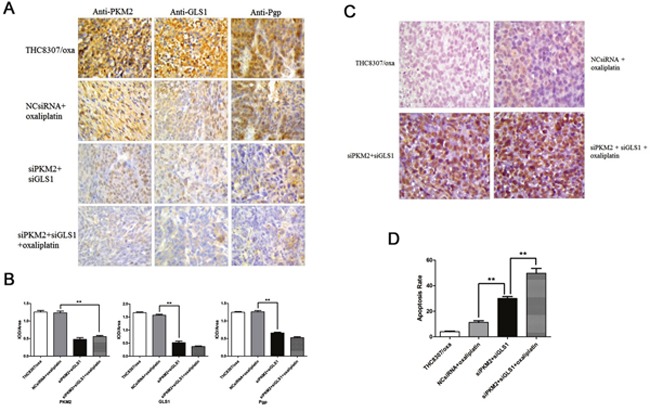
**(A-B)** Detection of PKM2, GLS1 and Pgp in xenograft by IHC analyses. PKM2, GLS1 and Pgp expression in xenograft was significantly reduced in the siPKM2 plus siGLS1 plus oxaliplatin group, as compared with that in the other three groups (**p < 0.01). **(C-D)** Apoptosis rate in four xenograft groups were 4.0 ± 0.5 (THC8307/Oxa), 9.0 ± 1.0 (NcsiRNA plus oxaliplatin), 29.0 ± 1.2 (siPKM2 plus siGLS1) and 50.0 ± 1.5 (siPKM2 plus siGLS1 plus oxaliplatin). apoptosis rate in the siPKM2 plus siGLS1 plus oxaliplatin group was significantly augmented, as compared with that in the other three xenograft groups (**p < 0.01).

IHC analyses were used to quantify the expression of PKM2, GLS1 and Pgp in xenograft. As shown in Figure 9A and 9B, PKM2 and GLS1 expression in xenograft was significantly reduced in group IV, which further reinforced the efficiency of knocking-down PKM2/GLS1 expression. Simultaneously, Pgp expression was also decreased in xenograft.

### Apoptosis rate in xenograft by Tunel assay

Tunel assay was employed to detect apoptosis rate in xenograft. As shown in Figure 9C and 9D, apoptosis rate in xenograft was 4.0 ± 0.5, 9.0 ± 1.0, 29.0 ± 1.2, and 50.0 ± 1.5 in group I, II, III and IV, respectively. Apoptosis rate in group IV was significantly augmented, as compared with that in the other three xenograft groups.

## DISCUSSION

Cancer is increasingly recognized as a metabolic disease featured by energy imbalance [19]. At the initial stage of tumor development, it is widely believed that mitochondria dysfunction occurs and ATP synthesis disrupts. As a consequence, tumor cells instead resort to aerobic glycolysis and glutamine metabolism as alternative ATP sources [20]. Aerobic glycolysis and glutaminolysis may contribute to the oxaliplatin-resistance of cancer cells through several manners, such as the increase of lactate production and export, the restriction on DNA oxidant drug efficiency, the inhibition of oxidative phosphorylation rate, and so on [21, 22]. Some investigators have proposed a workable strategy by focusing on restoring energy balance in order to delay tumor growth and reverse drug resistance [23, 24]. It is hence vital to detect and characterize some key modulators in aerobic glycolysis and glutaminolysis to elucidate the possible relationship between metabolic profiles and oxaliplatin-resistance in CRC [25]. Since PKM2 and GLS1 separately are two important rate-limiting enzymes in aerobic glycolysis and glutaminolysis [26, 27], we in the present study sought to investigate the possible contributory role of PKM2 and GLS1 expression in oxaliplatin-resistance CRC.

Our initial findings identified significantly elevated expression of PKM2 in the THC8307 cell line relative to the HCMEC cell line, at both mRNA and protein levels, which supported the notion that PKM2 might participate in the development of CRC. Further, we found that PKM2 expression was significantly higher in the THC8307 cell line than in the THC8307/Oxa cell line, the finding in agreement with that in the study by Martinez et al., who reported that PKM2 downregulation was involved in oxaliplatin-resistance in CRC [28, 29]. In the case of GLS1, it is believed to act as an oncogene and promote tumor growth [30, 31]. In the present study, GLS1 expression was significantly high in the THC8307 cell line compared with the HCMEC cell line, implying a contributory role of GLS1 in colorectal carcinogenesis. However, GLS1 expression was observed to be increased in the THC8307/Oxa cell line relative to the THC8307 cell line, antilogous for PKM2. To the best of our knowledge, this is the first report that has explored the association of GLS1 expression with oxaliplatin-resistance in CRC.

Considering the important role of PKM2 and GLS1 in CRC growth based on above evidence, we were inspired to investigate the influence of knocking-down PKM2/GLS1 expression in CRC cells. To fully address this issue, we have successfully constructed two siRNAs that were specifically targeted on PKM2 and GLS1, and found that both siRNAs could normally take effect without interference. After knocking-down PKM2/GLS1 expression, reduced IC50 was observed in both THC8307 and THC8307/Oxa cell lines, suggesting that PKM2/GLS1 downregulation could enhance the sensitivity for oxaliplatin and/or reverse drug-resistance in CRC cell lines. These observations agreed with the findings of previous studies that reported similar consequences of siPKM2 and siGLS1 in lung cancer and breast cancer cells [32, 33]. Then, a serial of malignant behaviors in CRC cells were inspected in the present study. After knocking-down PKM2/GLS1 expression, both THC8307 and THC8307/Oxa cell lines exhibited huge inhibitory efficiencies on cell formation ability, kinetic ability, migration ability, invasion ability and survival ability. These phenomena appear to be a clear explanation as to the anomaly of aerobic glycolysis and glutaminolysis, insufficiency of ATP synthesis, inequality of energy supply and generation in tumor cells [34].

As an energy-dependent transport protein, Pgp is involved in multi-drug resistance in cancer, and it further results in chemotherapy failure [35]. In the present study, immunofluorescence assay revealed that Pgp expression was significantly increased in the THC8307/Oxa cell line, but significantly decreased in both THC8307 and THC8307/Oxa cell lines after knocking-down PKM2/GLS1 expression. Moreover, PKM2/GLS1 downregulation was observed to inhibit Pgp expression, strengthen drug sensitivity in the THC8307 cell line and inverse oxaliplatin-resistance in the THC8307/Oxa cell line. These observations were in line with the findings of previous studies that reported that PKM2 can enhance chemosensitivity to cisplatin through interacting with the mTOR pathway in cervical cancer, and GLS1 downregulation could re-sensitize the Taxol-resistant breast cancer cells to Taxol [17, 26].

Anti-apoptosis represents another important mechanism for oxaliplatin-resistance [36]. As revealed by Annexin V/PI assay, apoptosis rate in the THC8307/Oxa cell line was lower than that in the THC8307 cell line, which can aid in explaining elevated oxaliplatin-resistance in the THC8307/Oxa cell line. Then, after knocking-down PKM2/GLS1 expression, apoptosis rate was significantly augmented in both THC8307 and THC8307/Oxa cell lines.

Finally, we examined the efficiency of knocking-down PKM2/GLS1 expression *in vivo*, and found that their knockdown exerted a significant tumor-inhibitory impact in xenograft. Correspondingly, Pgp expression was suspended, and apoptosis rate was elevated. There is strong agreement between *in vivo* and *in vitro* data, indicating the robustness of our findings.

A major drawback of the present study is that only one cell type (THC8307) was employed to investigate the relationship between PKM2/GLS1 expression and oxaliplatin-resistance in CRC. Bearing this drawback in mind, we will design a protocol for following investigations more elaborately and make results more reliable.

In summary, the present findings enriched our knowledge by demonstrating a significant association of two key rate-limiting enzymes, PKM2 and GLS1, with oxaliplatin-resistance in CRC. Moreover, differentiated expression of PKM2 and GLS1 in CRC cell lines can increase oxaliplatin-resistance by raising Pgp expression and inhibiting cell apoptosis, whereas in drug-resistance CRC cell lines, the knockdown of PKM2/GLS1 expression can restore oxaliplatin-sensitivity by degrading Pgp expression and inducing cell apoptosis. This present study deepens our understanding of molecular mechanisms underlying chemotherapy resistance of oxaliplatin in CRC cells by proposing that knockdown of PKM2/GLS1 expression may constitute a novel therapeutic strategy toward effective treatment for CRC in clinical practice.

## MATERIALS AND METHODS

### Cell lines and reagents

Highly differentiated CRC cell line (THC8307) and oxaliplatin-resistant CRC cell line (THC8307/Oxa) were purchased from the Saierbio Co., Ltd. (Saierbio, Tianjin, China). Normal human colon mucosa epithelial cell line (HCMEC) was kindly provided by Sun Yat-sen University Cancer Center. Oxaliplatin was purchased from the Sanofi-Avenitis (Sanofi-aventis US LLC, Bridgewater, NJ, USA). Anti-PKM2 was purchased from the Santa Cruz Co., Ltd. (Santa Cruz Biotechnology, CA, USA). The other antibodies were purchased from the Abcam (Abcam, Cambridge, UK). All study protocols followed standard guidelines, and were approved by the Human Research Ethics Committees of Sun Yat-sen University.

### Detection of PKM2/GLS1 expression

Both qRT-PCR and WB techniques in two CRC cell lines, THC8307 and THC8307/Oxa, were performed to quantify the PKM2/GLS1 expression, as previously described [37, 38]. The HCMEC cell line was used as the negative control.

### Small interfering RNA (siRNA) transfection of PKM2/GLS1

The GLS1/PKM2-siRNAs were chemically synthesized by the Sigma Chemical Co.,Ltd. (St. Louis, MO, USA). The sequences of siRNAs were as follows: for GLS1-siRNA1: 5′- GUU GAA AGA GUG UAU GGA UdT dT -3′ (forward) and 5′- AUC CAU ACA CUC UUU CAA CdT dT -3′ (reverse); for PKM2-siRNA2: 5′ -GAA UGA AUG UGG CUC GUC UdT dT -3′ (forward) and 5′- AGA CGA GCC ACA UUC AUU CdT dT -3′ (reverse). Then, the efficiency of siRNA transfection of PKM2/GLS1 in the THC8307 cell line was examined by WB technique. The NcsiRNA was used as the negative control.

### Detection of malignant behaviors in CRC cells

Colony formation, cell scratch, Transwell test, MTS test and IC50 calculation were performed in two CRC cell lines - THC8307 and THC8307/Oxa, before knocking-down PKM2/GLS1 expression. After its knockdown in the THC8307 cell line, all phenotype experiments were re-performed. All protocols were performed according to the methods described previously [39–41]. The HCMEC cell line was used as the negative control.

### Detection of Pgp expression

Pgp expression was detected by immunofluorescence assay before knocking-down PKM2/GLS1 expression. Both THC8307 and THC8307/Oxa cell lines were cultured in 6-well plates. After incubating and washing, the plate was blocked with 1% non-fat milk for 30 min, followed by administration of anti-Pgp antibody (1:50, abcam, ab10333). Then, a horseradish peroxidase (HRP) antibody was added (1:2000, Thermo Scientific, A16072). After knocking-down PKM2/GLS1 expression in the THC8307 cell line, Pgp expression was re-tested by immunofluorescence assay. The protocols were performed according to the methods described previously [42]. The HCMEC cell line was used as the negative control.

### Detection of apoptosis rate

Apoptosis rate of two CRC cell lines (THC8307 and THC8307/Oxa) was investigated by Annexin V/PI assay before knocking-down PKM2/GLS1 expression, as previously described [43]. After knocking-down PKM2/GLS1 expression in the THC8307 cell line, apoptosis assay was re-examined by Annexin V/PI assay. The HCMEC cell line was used as the negative control.

### CRC xenografts

Sixteen 4-week-old SCID mice with body weight of approximately 20 g were purchased from the Animal Center of Sun Yat-sen University, and they were raised under specific pathogen-free conditions. For tumor establishment, 2 × 10^7^ THC8307/Oxa cells per mL were washed twice with PBS, and were injected subcutaneously in a volume of 0.1 mL into the flank of mice. After inoculation, tumor-bearing mice were divided randomly into 4 treatment groups: THC8307/Oxa negative control (group I); NcsiRNA (100 μg/0.02 mL) plus oxaliplatin (5 mg/kg) (group II); siPKM2 (100 μg/0.02 mL) plus siGLS1 (100 μg/0.02 mL) (group III); siPKM2 (100 μg/0.02 mL) plus siGLS1 (100 μg/0.02 mL) plus oxaliplatin (5 mg/kg) (group IV). Animal experiments were conducted in accordance with the public health service policy, and they were approved by the Animal Care and Use Committees of Sun Yat-sen University. Mice bearing tumors were observed, and tumor size was measured once every 3 days using vernier caliper. At the end of the experiments (at the 28^th^ day), all animals were killed, and the tumors from each animal were removed and prepared [44].

### IHC analysis and tunel assay in xenografts

IHC analysis was performed as previously described to explore the expression of PKM2, GLS1 and Pgp [45]. Simultaneously, Tunel assay was conducted to investigate the apoptosis of xenografts, and the protocols were described previously [46].

### Statistical analyses

Quantitative data were described as means ± standard deviation. Two-group comparisons of quantitative data were completed by using the Student's t-test. Statistical significance was declared when the two-tailed p-value is 0.05 or less. Statistical analyses were accomplished with the SPSS Statistics Version 22.0 (IBM, Armonk, NY, USA).
